# Combining Bioactive Multifunctional Dental Composite with PAMAM for Root Dentin Remineralization

**DOI:** 10.3390/ma10010089

**Published:** 2017-01-22

**Authors:** Shimeng Xiao, Kunneng Liang, Michael D. Weir, Lei Cheng, Huaibing Liu, Xuedong Zhou, Yi Ding, Hockin H. K. Xu

**Affiliations:** 1 State Key Laboratory of Oral Diseases, West China Hospital of Stomatology, Sichuan University, Chengdu 610041, China; shimengxiao817@163.com (S.X.); kunnengliang@163.com (K.L.); chengleidentist@163.com (L.C.); zhouxd@scu.edu.cn (X.Z.); 2 Biomaterials and Tissue Engineering Division, Department of Endodontics, Periodontics and Prosthodontics, University of Maryland School of Dentistry, Baltimore, MD 21201, USA; mweir@umaryland.edu; 3 L.D. Caulk Division, Dentsply Sirona Restorative, Milford, DE 19963, USA; Huaibing.Liu@dentsplysirona.com; 4 Center for Stem Cell Biology & Regenerative Medicine, University of Maryland School of Medicine, Baltimore, MD 21201, USA; 5 Department of Mechanical Engineering, University of Maryland, Baltimore County, MD 21250, USA

**Keywords:** root dentin, root caries, multifunctional composite, acid challenge, calcium phosphate nanoparticles, poly (amido amine)

## Abstract

*Objectives*. The objectives of this study were to: (1) develop a bioactive multifunctional composite (BMC) via nanoparticles of amorphous calcium phosphate (NACP), 2-methacryloyloxyethyl phosphorylcholine (MPC), dimethylaminohexadecyl methacrylate (DMAHDM) and nanoparticles of silver (NAg); and (2) investigate the effects of combined BMC + poly (amido amine) (PAMAM) on remineralization of demineralized root dentin in a cyclic artificial saliva/lactic acid environment for the first time. *Methods*. Root dentin specimens were prepared and demineralized with 37% phosphoric acid for 15 s. Four groups were prepared: (1) root dentin control; (2) root dentin with BMC; (3) root dentin with PAMAM; (4) root dentin with BMC + PAMAM. Specimens were treated with a cyclic artificial saliva/lactic acid regimen for 21 days. Calcium (Ca) and phosphate (P) ion concentrations and acid neutralization were determined. The remineralized root dentin specimens were examined via hardness testing and scanning electron microscopy (SEM). *Results*. Mechanical properties of BMC were similar to commercial control composites (*p* = 0.913). BMC had excellent Ca and P ion release and acid-neutralization capability. BMC or PAMAM alone each achieved slight mineral regeneration in demineralized root dentin. The combined BMC + PAMAM induced the greatest root dentin remineralization, and increased the hardness of pre-demineralized root dentin to match that of healthy root dentin (*p* = 0.521). *Significance*. The excellent root dentin remineralization effects of BMC + PAMAM were demonstrated for the first time. BMC + PAMAM induced effective and complete root dentin remineralization in an acid challenge environment. The novel BMC + PAMAM method is promising for Class V and other restorations to remineralize and protect tooth structures.

## 1. Introduction

Dental caries is a prevalent and worldwide burden [1,2,3,4]. Acid-producing bacteria feed on fermentable carbohydrates and produce organic acids as byproducts [5]. The acids dissolve hydroxyapatite minerals, leading to caries lesions [6]. Remineralization is the natural repair process for caries lesions in vivo [7]. However, natural remineralization in vivo can only overcome a certain level of caries (acid) challenges. When bacterial acid challenge is severe, natural remineralization is insufficient to halt or reverse the caries process [7]. Therefore, the development of novel antibacterial and remineralization dental materials is needed to help combat caries and protect tooth structures.

Composites are widely used as filling materials due to their esthetics and direct-filling capabilities [8,9,10]. However, previous studies showed that composites are challenged with the accumulation of more biofilms and plaque than other restorative materials [11,12], which could result in secondary caries at the composite-tooth margins. Furthermore, the prevalence and severity of tooth root caries increases with aging, from 7% among young people to 56% in seniors of ≥75 years of age [13]. This is a growing public health issue due to the rapid increase in the elderly population along with increases in tooth retention in seniors [14,15]. Gingival recession due to aging, periodontal disease or traumatic tooth-brushing habits can increase the susceptibility to root caries [16]. Furthermore, low salivary flow in seniors and patients with dry mouths further contribute to biofilm and plaque buildup and the occurrence of root caries. Root caries can be treated with Class V restorations. However, restorations with subgingival margins are difficult to clean, which in turn could enhance the development of periodontitis and the loss of the tooth’s attachment [10]. Therefore, there is a need to develop a bioactive Class V composite to combat secondary caries and root caries.

To inhibit caries, nanoparticles of amorphous calcium phosphate (NACP) were incorporated into composite with calcium (Ca) and phosphate (P) ion release [17], which could remineralize tooth lesions [18]. The NACP nanocomposite was “smart” to increase the ion release at cariogenic pH when these ions were most needed, and could rapidly neutralize acid challenges to raise a cariogenic pH of 4 to a safe pH of 6 [19]. In addition, quaternary ammonium methacrylates (QAMs) have been incorporated into resins to achieve antibacterial functions to reduce biofilm acids [20,21,22,23]. The antibacterial activity of QAMs was shown to increase with increasing the alkyl chain length (CL) from 5 to 16 for the ammonium groups [24,25]. A recent study demonstrated that a composite containing dimethylaminohexadecyl methacrylate (DMAHDM) with CL of 16 had a strong antimicrobial function [25].

Protein-repellent resins represent another approach to biofilm reduction. Salivary protein coating on the resin could decrease the efficacy of “contact-inhibition” via QAMs [26]. Therefore, it would be desirable to develop protein-repellent functions that can reduce protein adsorption and enhance contact-inhibition. A previous study showed 2-metha-cryloyloxyethyl phosphorylcholine (MPC) could resist protein adsorption and bacterial adhesion [27,28]. Recently, MPC was incorporated into dental resins showing strong protein-repellent properties and inhibition against oral bacteria [22,29,30]. Furthermore, resins containing silver (Ag) nanoparticles (NAg) were reported to also have a potent antibacterial activity [31,32,33]. Incorporating NAg with small particle sizes and a high surface area into resin achieved a strong antimicrobial function [34,35]

To remineralize tooth lesions, poly (amido amine) (PAMAM) dendrimer was important for remineralization [36,37,38]. Several types of PAMAM dendrimers were used as nucleation templates to induce tooth remineralization in tooth structures [36,37,38]. PAMAM is an excellent nucleation template and can rapidly absorb Ca and P ions to cause remineralization [36,37,38]. However, these aforementioned approaches have not been combined to develop a bioactive Class V composite for treating root caries.

In our recent study, a Class V composite with NACP, DMAHDM and MPC was developed and effectively inhibited biofilms of periodontal pathogens [10]. However, the remineralization capability of this new composite was not investigated. In another study, the combined PAMAM plus NACP composite approach was shown to induce effective dentin remineralization in a cyclic artificial saliva/lactic acid regimen [39]. The combination of PAMAM and NACP achieved triple benefits of excellent nucleation template, high concentration of Ca and P ions, and acid-neutralization. However, to date, there has been no report on the remineralization effects of PAMAM + NACP composite on tooth root dentin. Furthermore, there has been no report on the combination of PAMAM, NACP, DMADHM, MPC and NAg to achieve strong antibacterial and remineralization capabilities.

Therefore, the objectives of the present study were to: (1) develop a new bioactive multifunctional composite (BMC) with NACP, DMADHM, MPC and NAg for Class V restorations; and (2) investigate the effects of BMC, PAMAM, and BMC + PAMAM on root dentin remineralization for the first time. It was hypothesized that: (1) BMC would greatly increase Ca and P concentrations, neutralize acid and promote root dentin remineralization in a cyclic artificial saliva/acid treatment; (2) PAMAM would remineralize the demineralized root dentin in cyclic artificial saliva/lactic acid regimen; (3) The combined BMC + PAMAM approach would induce the greatest root dentin remineralization among the test groups.

## 2. Materials and Methods

### 2.1. Preparation of Bioactive Multifunctional Composite (BMC)

Ethoxylated bisphenol A dimethacrylate (EBPADMA, Sigma-Aldrich, St. Louis, MO, USA) and pyromellitic dianhydride glycerol dimethacrylate (PMDGDM, Esstech, Essington, PA, USA) were mixed at 1:1 mass ratio, following a previous study [40]. It was rendered light-curable with 0.2% camphorquinone and 0.8% ethyl 4-N,N-dimethylaminobenzoate (all by mass, unless noted otherwise) [40]. This EBPADMA-PMDGDM resin is referred to as EBPM.

Silver 2-ethylhexanoate (Strem, Newburyport, MA, USA) of 0.12 g was dissolved into 0.88 g of 2-(tert-butylamino)ethyl meth-acrylate (TBAEMA, Sigma-Aldrich, St. Louis, MO, USA) [41]. TBAEMA could facilitate Ag-salt dissolution in resin [42]. Of this solution, 1% was added to EBPM resin to form EBPM-NAg resin, yielding 0.12% of Ag salt in resin following previous studies [41,42].

MPC was obtained commercially (Sigma-Aldrich, St. Louis, MO, USA) which was synthesized using a method reported previously [28]. MPC is a methacrylate with a phospholipid polar group in the side chain [28]. MPC was incorporated into the EBPM-NAg resin, yielding a 3% MPC content in the final composite.

DMAHDM was synthesized using a modified Menschutkin reaction in which a tertiary amine group was reacted with an organohalide [43]. Briefly, 10 mmol of 2-(dimethylamino)ethyl methacrylate (DMAEMA, Sigma-Aldrich, St. Louis, MO, USA) and 10 mmol of 1-bromohexadecane (BHD, TCI America, Portland, OR, USA) were combined with 3 g of ethanol in a 20 mL scintillation vial. The vial was stirred at 70 °C for 24 h. The solvent was then removed via evaporation, yielding DMAHDM as a clear, colorless, and viscous liquid [44]. DMAHDM was mixed with EBPM-NAg-MPC resin, yielding a DMAHDM mass fraction of 3% in the final composite.

NACP were synthesized using a spray-drying technique as previously described [17]. Briefly, calcium carbonate and dicalcium phosphate anhydrous were dissolved in acetic acid to produce Ca and P concentrations of 8 mmol/L and 5.333 mmol/L, respectively, thus yielding a Ca/P molar ratio of 1.5, the same as that for ACP [Ca_3_(PO_4_)_2_]. This solution was sprayed into a heated chamber of the spray-drying machine. An electrostatic precipitator was used to collect the dried particles. This produced NACP with a mean particle size of 116 nm [17]. Barium boroaluminosilicate glass particles with a median size of 1.4 μm were used as a co-filler (Caulk/Dentsply, Milford, DE, USA), which was silanized with 4% 3-methacryloxypropyltrimethoxysilane and 2% npropylamine [17]. NACP was incorporated into the EBPM-NAg-MPC-DMADHM resin at a NACP filler mass fraction of 30% in the final composite, while the glass particle filler level was 35%. The mixed composite paste was placed into a mold of 2 mm × 2 mm × 25 mm, and light-cured (Triad 2000, Dentsply, York, PA, USA) for 1 min on each open side. In the composite, the final mass fractions of MPC, DMADHM, NACP, glass filler and EBPM-NAg resin matrix were 3%, 3%, 30%, 35% and 29%, respectively. Two experimental composites were fabricated:

(1) Antibacterial and remineralizing composite: 3% MPC + 3% DMAHDM + 30% NACP + 35% glass fillers + 29% EBPM-NAg resin matrix (referred to as “bioactive multifunctional composite”, or “BMC”);
(2) Experimental control without antibacterial agents: 30% NACP + 35% glass fillers + 35% EBPM resin matrix (referred to as “EBPM + NACP” composite).

In addition, two commercial composites served as controls in mechanical testing. Heliomolar is a nanocomposite that contained silica and ytterbium-trifluoride fillers with particle sizes of 40–200 nm at a filler level of 66.7%. Heliomolar was selected because it is indicated for Class I and II restorations as well as Class V restorations for root caries, Heliomolar served as control I. Another nanocomposite (Renamel Microfill, Cosmedent, Chicago, IL, USA) served as control II. It consisted of fillers of 40 nm to 200 micron at 60% filler level by volume in a multifunctional resin of diurethane dimethacrylate and butanediol dimethacrylate. Renamel was selected because it is indicated for Class III, IV, and V restorations. All specimens were light-cured in the same manner as described above.

### 2.2. PAMAM Synthesis

PAMAM dendrimers were synthesized as described previously [45]. Briefly, the divergent synthesis of PAMAM dendrimers included a two-step interactive sequence to produce amine-terminated structures. Iterative sequencing involved alkylation with methyl acrylate (MA) followed by amidation with excess 1,2-ethylenediamine (EDA). The alkylation step produced ester-terminated intermediates that were called “half-generations”. The second step involved amidation of the ester-terminated intermediates with a large excess of EDA to produce amine terminated intermediates, which were called “full-generations”. The first and second generations are linear molecules, while the third generation is a sphere molecule with more functional groups, which ensures that it could absorb more Ca and P ions during remineralization. The present study used the third generation PAMAM-NH_2_, which was obtained commercially (Chenyuan Dendrimer Tech., Weihai, China). Previous studies showed that PAMAM-NH_2_ effectively remineralized the demineralized dentin [46]. In this article, the term “PAMAM” refers to the third generation PAMAM-NH_2_. The PAMAM solution was prepared by dissolving 100 mg of PAMAM powder in 100 mL of distilled water to achieve a concentration of 1 mg/mL, following a previous study [37].

### 2.3. Mechanical Testing

All composite specimens were stored at 37 °C for 24 h, and then fractured in three-point flexure with a 10 mm span at a crosshead-speed of 1 mm/min on a computer-controlled Universal Testing Machine (5500R, MTS, Cary, NC, USA) [17]. Flexural strength (S) was calculated as: *S* = 3*P*_max_*L*/(2*bh*^2^), where *P* is the fracture load, *L* is span, *b* is specimen width and *h* is thickness. Elastic modulus (*E*) was calculated as: *E* = (*P*/*d*)(*L*^3^/(4*bh*^3^)), where load *P* divided by displacement *d* is the slope in the linear elastic region [17].

### 2.4. Preparation of Root Dentin Specimens

Caries-free and single-rooted human permanent premolars were collected from the dental school clinics following a protocol approved by the University of Maryland Institutional Review Board. Teeth were disinfected in a 0.005% promodyne solution for 4 h and stored at 4 °C in distilled water. Root dentin squares of 4 mm × 4 mm × 1 mm were prepared by cutting parallel to the long axis of the tooth 2 mm below the cemento-enamel junction using a diamond blade (Buehler, Lake Bluff, IL, USA). The root dentin specimens were acid-etched with 37% phosphoric acid for 15 s to create demineralization, following a previous study [47]. This acid etching method was used in order to investigate the remineralization of demineralized dentin with a large amount of exposed collagen fibrils and prevent secondary caries at the bonded dentin-restoration interface [47]. The demineralized root dentin specimens were sonicated in distilled water for 10 min to remove any debris and then stored at 4 °C in phosphate-buffered saline (PBS, pH 7.4) before use.

### 2.5. Root Dentin Remineralization in an Acid Challenge Environment

The demineralized tooth root dentin specimens were randomly divided into four groups:

(1) Control group. Each demineralized root dentin specimen was coated with 100 μL of distilled water, and then air dried to serve as a negative control [37].
(2) BMC group. Each demineralized root dentin specimen was placed in contact with three BMC bars of 2 mm × 2 mm × 12 mm [35]. Three bars were used because, when subsequently immersed in 1 mL solution; this would yield a composite volume/solution volume ratio of 0.14/1, the same as that in a previous study [35].
(3) PAMAM group. Each demineralized root dentin specimen was coated with 100 μL of the PAMAM solution which was kept on dentin for 1 h to ensure that PAMAM macromolecules were immobilized on root dentin, and then the specimen was rinsed with water to remove any loose PAMAM [37].
(4) BMC + PAMAM group. Each demineralized root dentin specimen was first coated with 100 μL of PAMAM solution, and then three BMC bars of 2 mm × 2 mm × 12 mm were placed on root dentin specimen.

Six specimens were tested for each group (*n* = 6). A 1.5 mL conical vial was used to store each sample which was completely immersed in 1 mL of a solution below. Artificial saliva solution was prepared by dissolving, in distilled water, 1.5 mmol/L CaCl_2_, 0.9 mmol/L KH_2_PO_4_, 130 mmol/L KCl, 1.0 mmol/L NaN_3_ and 20 mmol/L 4-(2-hydroxyethyl)-1-piperazineethanesulfonic acid (HEPES), and adjusting to pH 7.0 with KOH (1 mmol/L) [48]. Artificial saliva is a solution to mimic the human saliva, which can supply Ca and P ions for the remineralization [48]. In addition, a sodium chloride (NaCl) solution (133 mmol/L) was buffered to pH 4 with 50 mmol/L lactic acid to simulate accelerated cariogenic conditions (referred to as “lactic acid solution”) [17]. Each day, each specimen of the aforementioned four groups was immersed in 1 mL of fresh artificial saliva for 23 h, and then in 1 mL of lactic acid solution for 1 h at 37 °C, following previous studies [18]. This was repeated for 21 days.

### 2.6. Ca and P Ion Concentrations Measurement

At 1, 3, 5, 7, 10, 14 and 21 days, the Ca and P ion concentrations in the lactic acid and artificial saliva solutions were measured. At each time, the 1 mL solution was removed and replaced by fresh solution. The collected solution was analyzed for Ca and P ion concentrations via a spectrophotometric method (DMS-80 UV-visible, Varian, Palo Alto, CA, USA) using known standards and calibration curves, following previous studies [49].

### 2.7. Acid Neutralization

At 1, 3, 5, 7, 10, 14 and 21 days, the pH values of lactic acid solutions of the four groups were measured. Every day, each sample was immersed in the artificial saliva solution for 23 h, and in the lactic acid solution for 1 h. During that 1 h in lactic acid solution, the pH was monitored with a combination pH electrode (Orion, Cambridge, MA, USA). The pH in artificial saliva was also measured.

### 2.8. Dentin Hardness Measurement

Dentin hardness was related to the amount of minerals in the dentin structure; hence, surface hardness was usually measured to evaluate the remineralization extent [27]. The hardness of dentin was measured for the four groups after 21 days. A hardness tester (Tukon 2100B, Instron, Canton, MA, USA) was used with a Vickers diamond indenter at a load of 20 g and a dwell time of 10 s [27]. Six indentations were made in each dentin, and six dentin specimens were tested for each group. In addition, hardness values of sound healthy dentin without acid treatment, and dentin after acid-etching but without the 21 days of immersion treatment, were also measured as comparative controls.

### 2.9. Scanning Electron Microscopic (SEM) Examination

After 21 days, all root dentin squares were removed from the solutions to examine whether there were minerals regenerated in the demineralized root dentin surface and in the dentinal tubules. The root dentin square was cut with a diamond saw (Buehler, Lake Bluff, IL, USA) into two halves along the midline. One half was used for observing the occlusal section (the observed surface was perpendicular to the tubule axis). The other half was used for observing the longitudinal section (the observed surface was parallel to the tubule axis). The root dentin samples were sonicated in water for 10 min to remove the debris caused by the cutting. They were then immersed in 1% glutaraldehyde in PBS for 4 h at 4 °C. They were rinsed with PBS and subjected to graded ethanol dehydrations, and then rinsed with 100% hexamethyldisilazane [19]. Then the root dentin samples were sputter-coated with gold and examined via scanning electron microscopy (SEM, JEOL 5300, Peabody, MA, USA).

### 2.10. Statistical Analysis

All data were checked for normal distribution with the Kolmogorov-Smirnov test. For power analysis and specimen size, our previous and preliminary studies indicated typical standard deviations of about 10%–30% of the mean. The differences between control and remineralizing groups on Ca and P ions and dentin hardness are expected to be at least 100%. A 5% confidence level and a power to detect a treatment effect of 95% let to the use of *n* = 6. One-way and two-way analyses of variance (ANOVA) were performed to detect the significant effects of the variables. Tukey’s multiple comparison tests were used at a *p* value of 0.05.

## 3. Results

**Figure 1:**
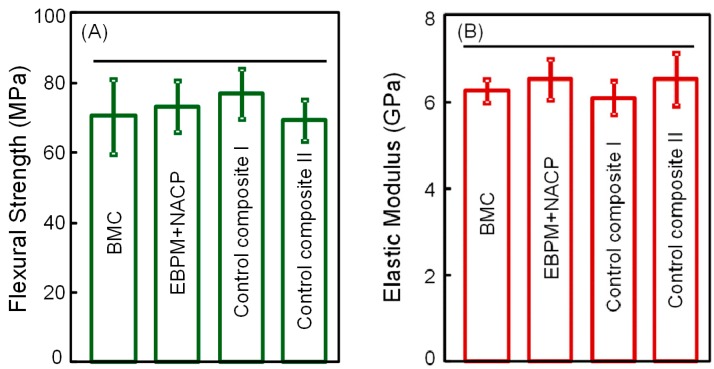
Mechanical properties of composites: (**A**) Flexural strength, and (**B**) elastic modulus (mean ± SD; *n* = 6). Bioactive multifunctional composite (BMC) had flexural strength and elastic modulus similar to EBPADMA-PMDGDM (EBPM) + nanoparticles of amorphous calcium phosphate (NACP) composite and commercial control composites. Horizontal line indicates values that are not significantly different (*p* = 0.875).

The mechanical properties of composites are plotted in Figure 1A (flexural strength), and Figure 1B (elastic modulus) (mean ± SD; *n* = 6). Flexural strength and elastic modulus of BMC were not significantly different from those of commercial control composites (*p* = 0.913), suggesting that BMC could potentially be used in restorations where Heliomolar and Renamel are used, with the benefits of containing MPC and DMAHDM for antibacterial activity, and NACP for remineralization.

**Figure 2:**
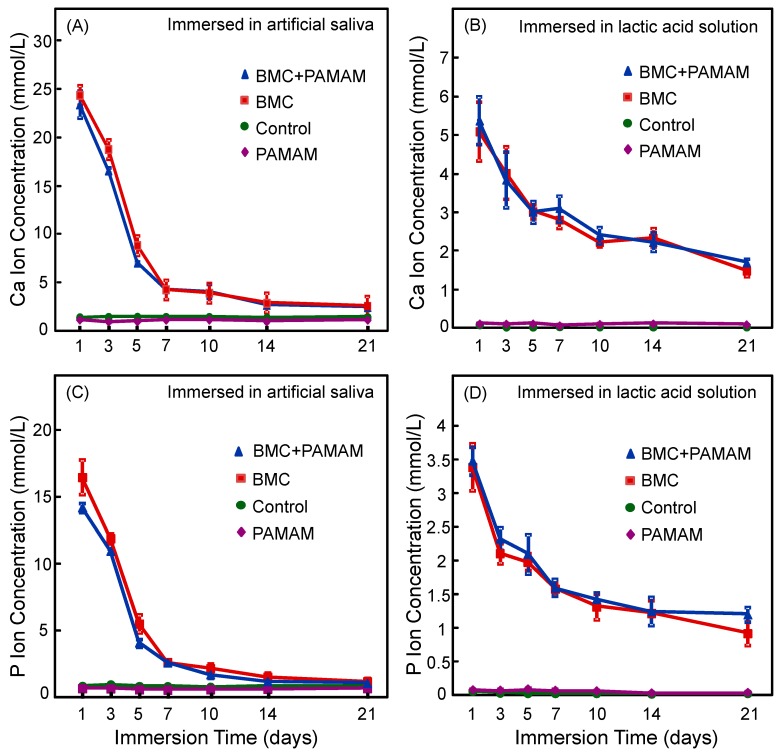
Ca and P ion concentrations (mean ± SD; *n* = 6). (**A**,**C**) Ca and P concentrations in artificial saliva, (**B**,**D**) Ca and P ion concentrations in lactic acid. The BMC group and BMC + poly (amido amine) (PAMAM) group had much higher Ca and P ion concentrations than PAMAM and control groups. For BMC and BMC + PAMAM groups, the ion concentrations decreased with time because every day a fresh solution was used to immerse the specimens.

Ca and P ion concentrations for the four groups are plotted in Figure 2A,C (artificial saliva), and Figure 2B,D (lactic acid solution) (mean ± SD; *n* = 6). For artificial saliva, the Ca and P concentrations of BMC and BMC + PAMAM groups were significantly higher than PAMAM and control groups (*p* = 0.009). For lactic acid solution, the Ca and P ion concentrations were nearly zero for PAMAM and control groups. In contrast, the Ca and P concentrations were also higher for BMC and BMC + PAMAM groups due to ion release from BMC nanocomposite. While ion concentrations decreased from 1 to 21 days due to the change of a fresh solution each day, BMC and BMC + PAMAM groups at 21 days still had significantly higher ion concentrations than PAMAM and control groups (*p* = 0.014), indicating continued ion release from BMC nanocomposite.

**Figure 3:**
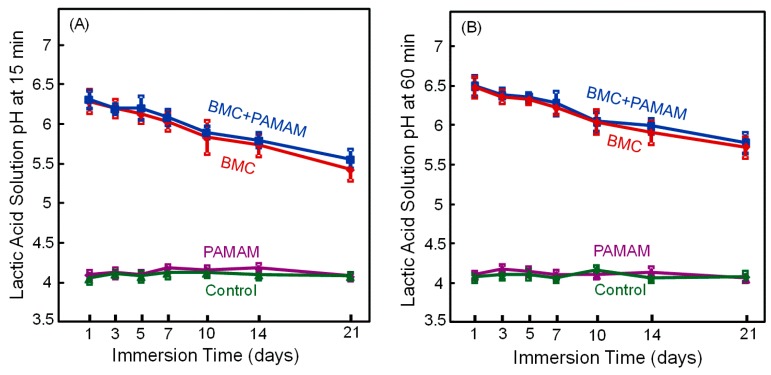
Acid neutralization. The pH of lactic acid solution was measured after the specimen was immersed for: (**A**) 15 min, and (**B**) 60 min (mean ± SD; *n* = 6). This was done for 21 days. The BMC group and BMC + PAMAM group neutralized the acid and raised the pH. PAMAM and control groups had no acid neutralization and the pH stayed near 4.

Specimens of the four groups were immersed in artificial saliva at pH 7 for 23 h, and in pH 4 lactic acid solution for 1 h every day. The pH values of the lactic acid are plotted in Figure 3, with pH being measured (A) at 15 min, and (B) at 60 min, after the specimen was immersed in the solution (mean ± SD; *n* = 6). For PAMAM and control group, the pH stayed at 4. In contrast, for BMC and BMC + PAMAM groups, at 1 day, the pH was increased to 6.3 at 15 min and 6.5 at 60 min. The pH showed a slight decrease with increasing the number of days because a fresh pH 4 solution was used each day to immerse the sample. At 21 days, the pH reached 5.4 at 15 min, and 5.7 at 60 min. These pH values are much higher than the pH 4 of the groups without BMC (*p* = 0.005).

**Figure 4:**
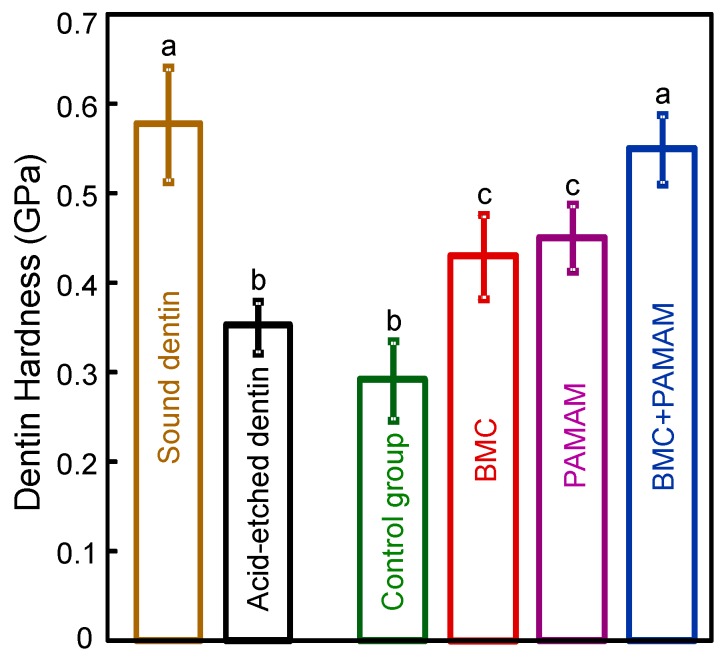
Hardness of root dentin. Healthy untreated root dentin and acid-etched root dentin were included for comparison. Four groups were measured after 21 days of artificial saliva/lactic acid regimen treatment: Control, BMC group, PAMAM group, and BMC + PAMAM group. Dissimilar letters indicate significantly different values (*p* = 0.022).

The root dentin hardness values are plotted in Figure 4 (mean ± SD; *n* = 6). The hardness of healthy sound root dentin was 0.58 ± 0.06 GPa. After demineralization with 37% phosphoric acid, root dentin hardness decreased to 0.35 ± 0.03 GPa (*p* = 0.015). After 21 days of the cyclic artificial saliva/lactic acid regimen, root dentin hardness of control group decreased to 0.29 ± 0.05 GPa. In contrast, BMC or PAMAM alone yielded greater dentin hardness, which was 0.43 ± 0.05 and 0.45 ± 0.04 GPa, respectively. BMC + PAMAM had the greatest root dentin hardness of 0.55 ± 0.04 GPa, which approached that of sound root dentin (*p* = 0.521).

**Figure 5:**
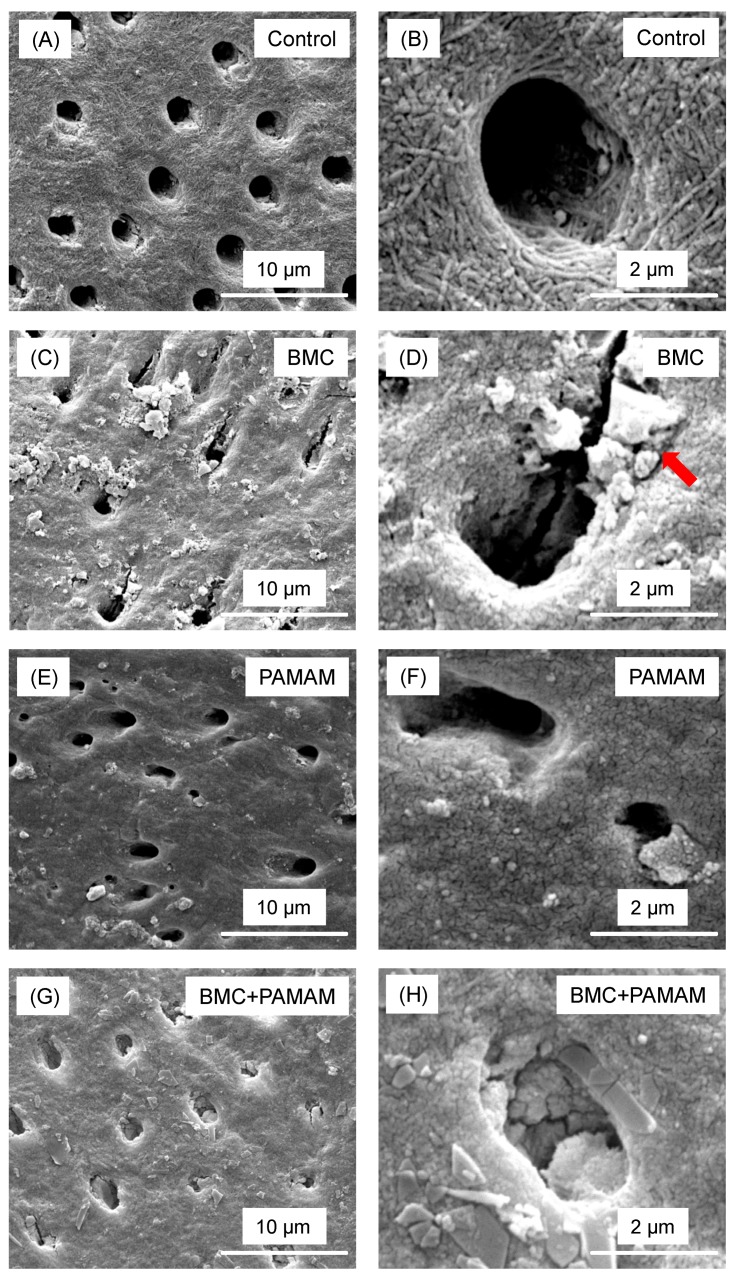
Representative SEM images of demineralized root dentin surface perpendicular to tubule axis after 21 days cyclic artificial saliva/lactic acid regimen: (**A**,**B**) control group, (**C**,**D**) BMC group, (**E**,**F**) PAMAM group, and (**G**,**H**) BMC + PAMAM group. Left column is at a lower magnification. Right column is at a higher magnification. Exposed collagen fibrils were detected in (**A**,**B**). (**C**–**F**) showed that regenerated minerals precipitated on the root dentin surface. Arrows showed minerals partially occluding the dentinal tubules. In (**G**,**H**), the dentin was covered by the remineralized mineral crystals.

Representative SEM micrographs of root dentin sections perpendicular to the tubules are shown in Figure 5. For control group, there were no minerals regenerated on the demineralized root dentin surface. BMC and PAMAM showed moderate remineralization, characterized by minerals precipitated in root dentin surfaces. In contrast, BMC + PAMAM showed the greatest remineralization, with much greater mineral growth than PAMAM or BMC alone.

**Figure 6:**
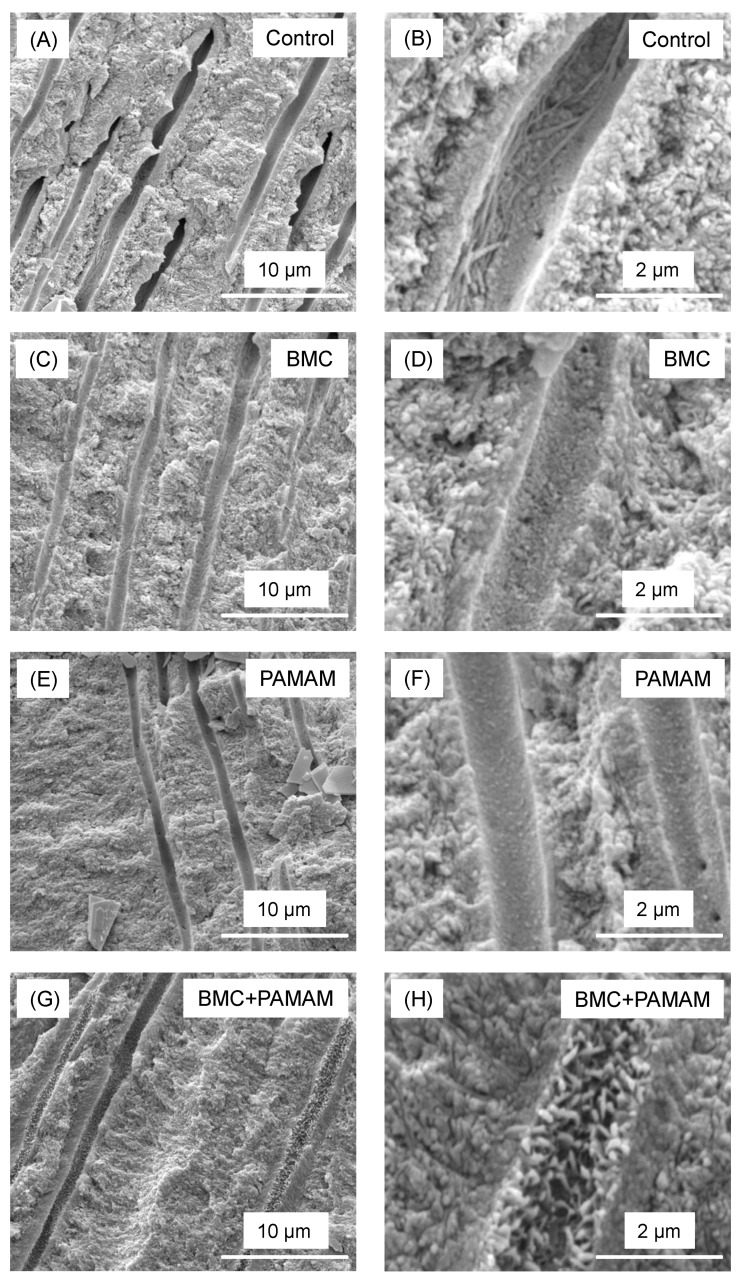
Representative scanning electron microscopy (SEM) images of demineralized root dentin subsurface cross-section parallel to tubule axis after 21 days cyclic artificial saliva/lactic acid regimen: (**A**,**B**) control; (**C**,**D**) BMC group; (**E**,**F**) PAMAM group; and (**G**,**H**) BMC + PAMAM. Left column is a lower magnification. Right column is a higher magnification. Exposed collagen fibrils were detected in (**A**,**B**). (**C**–**F**) show that minerals precipitated in tubules. In (**G**,**H**), the dentinal tubules were occluded by a great amount of the remineralized minerals.

Representative SEM micrographs of root dentin parallel to the tubules are shown in Figure 6. All pictures were taken on root dentin cross-sections in a subsurface region of 2–30 μm beneath the external surface. For control group, empty dentinal tubules were clearly observed. In contrast, for BMC and PAMAM groups, minerals were deposited in the dentinal tubules. The greatest mineral regeneration in root tubules was achieved via BMC + PAMAM.

## 4. Discussion

The present study developed a bioactive multifunctional composite (BMC) containing NAg, MPC, DMAHDM and NACP, and combined this composite with PAMAM to combat tooth caries for the first time. This approach achieved excellent root dentin remineralization. As an example, for Class V restorations, the new bioactive composite and the combination with PAMAM are potentially applicable to other types of restorations. Previous studies showed that a composite containing MPC, DMAHDM and NACP was effective to inhibit periodontitis-related pathogens for subgingival Class V restorations [10]. The new method in the present study remineralized the demineralized root dentin in a cyclic artificial saliva/lactic acid regimen. The hypotheses were proven that adding NAg, MPC, DMAHDM and NACP did not compromise the mechanical properties, and that BMC combined with PAMAM was the most effective method to inhibit demineralization and promote remineralization for tooth root dentin.

A previous study showed that a novel NACP composite containing QADM and NAg had strong antibacterial capability that was maintained after 12 months of water-aging [34], and the mechanical properties of the bioactive composite matched those of commercial control composites without antibacterial properties [34]. BMC may also sustain its good mechanical properties and antibacterial properties. First, MPC and DMAHDM were immobilized in the resin; they would not be released with time, which would ensure its sustained antibacterial capability. Second, NAg would be released to the surrounding environment to improve antibacterial property at the early stage as it could lead to bacterial cell death [34]. However, the release of NAg should not significantly affect BMC’s mechanical properties since the NAg mass fraction was only 0.12% in the EBPM-NAg resin. Furthermore, Ca and P ion release from NACP would indeed leave nano-voids in the restoration. However, the BMC can be repeatedly recharged with Ca and P ions [40], which would fill these voids and potentially maintain its mechanical properties. Further studies are needed to investigate whether the mechanical properties could be maintained in the long term.

Tooth root caries is a growing public health issue due to the rapid increase in the elderly population coupled with increases in tooth retention in seniors [14,15], Gingival recession can increase the susceptibility to root caries [16]. Furthermore, low salivary flow further contributes to biofilm and plaque buildup and the occurrence of root caries. However, Class V restorations with subgingival margins may be difficult to clean and this in turn could enhance the development of periodontitis and the loss of the tooth's attachment [10]. Moreover, the process of dental caries is that acid-producing bacteria feed on fermentable carbohydrates and produce organic acids as byproducts [5], and these acids dissolve hydroxyapatite minerals to form caries [6]. Glass ionomer cements are often used in Class V restorations due to their good biocompatibility with dental pulp tissues and fluoride release. However, previous studies indicated that a commercial resin-modified glass ionomer was not potent enough to inhibit bacterial growth and biofilm formation [22]. In addition, glass ionomer cement was relatively less esthetic for anterior teeth and cervical lesions [50]. Therefore, the present study focused on developing an antibacterial and remineralizing composite for Class V restorations, because it is highly desirable to develop a novel multifunctional composite for Class V restoration to achieve remineralization to strengthen and protect the root structures, while being able to inhibit cariogenic and periodontitis-related pathogens.

A previous study showed that 3% DMAHDM + 3% MPC in the composite achieved substantial reduction in biofilm growth, metabolic activity and polysaccharide production [10]. Biofilm CFU counts were reduced by nearly four orders of magnitude for four species of periodontal pathogens [10]. However, the remineralization ability of this composite was not demonstrated. Another previous study showed that PAMAM + NACP composite induced great coronal dentin remineralization in a cyclic artificial saliva/lactic acid solution regimen [39]. The combination of NACP with PAMAM achieved triple benefits of excellent nucleation templates, high Ca and P ion concentrations, and acid-neutralization [39]. However, the remineralization of root dentin had not been investigated in previous studies.

It is highly promising that combination of NAg, MPC, DMAHDM, NACP, and PAMAM could promote further remineralization. First, salivary proteins adsorbed onto the material surface in the oral environment provide the medium for the attachment of bacteria [51]. Therefore, making a protein-repellent composite would help repel bacterial attachment. MPC polymer has been shown to have excellent resistance to protein adsorption and bacterial adhesion [52]. MPC is highly hydrophilic and there is an abundance of free water but no bound water in the hydrated MPC polymer [27,28]. The large amount of free water around the phosphorylcholine group is considered to help detach proteins [52]. MPC polymer can repel protein adsorption and inhibit bacterial adhesion, thus reducing biofilm growth. Second, QAMs and NAg were both shown to be antibacterial, and the mode of antibacterial action for QAMs is suggested to be contact-inhibition [53]. The positively charged quaternary amine N+ interacts with the negatively charged cell membrane of bacteria, leading to membrane disruption and cytoplasmic leakage [53]. MPC could repel the proteins from covering the composite surface, leading to direct contact of the composite surface with bacteria, thus enhancing the contact-killing efficacy. NAg is another effective antibacterial agent which has a strong toxicity to a wide range of microorganism [54]. Its antimicrobial mechanism appears to involve Ag ions interacting with and inactivating the vital enzymes of bacteria, causing the DNA in the bacteria to lose its replication ability, leading to cell death [34,54]. A previous study demonstrated that adding dual agents (QADM + NAg) in the same composite resulted in a significantly stronger antibacterial capability than using QADM or NAg alone [34]. The combined use of MPC, DMAHDM and NAg greatly reduced biofilm growth and acid production, reducing biofilm CFU by three orders of magnitude, compared to commercial control [24]. In addition, NACP nanocomposite could greatly increase the Ca and P ion release at cariogenic low pH, when these ions were most needed to inhibit caries [17]. Furthermore, NACP nanocomposite was a promising remineralization agent, which had an enamel lesion remineralization efficacy that was four-fold that of a commercial fluoride-releasing composite [55]. The NACP nanocomposite also reduced caries at the enamel-composite margins to 1/3 that of a control composite under oral biofilms in a human in situ study [56,57]. Moreover, PAMAM could bind to demineralized dentin by electrostatic interactions and size-exclusion features of collagen fibrils [37]. During remineralization, PAMAM can attract Ca ions through Ca complexes via large numbers of amine groups on the external surface and amide groups in its branches [37]. Therefore, these previous studies justified the combined use of these agents (NAg, MPC, DMAHDM, NACP, and PAMAM) in the present study for maximum benefits in defeating dental caries. The present study focused on the development of the BMC and the remineralization ability on tooth root dentin. The antibacterial properties of this new combined approach will be investigated in a separate paper.

Most previous remineralization studies were performed at a neutral pH solution [58,59]. However, acids produced by oral biofilms or derived from acidic food can lower the local pH to 4.5 or 4 [60]. Hence, acid challenge should be taken into account in a remineralization study. In the present study, we employed a pH-cycling model to simulate the oral environment, and artificial saliva at pH 7 for remineralization and lactic acid at pH 4 for acid challenge were used [48]. Artificial saliva is the most common remineralization solution, and lactic acid accounts for the majority of biofilm acids [48]. In addition, a pH value of 4 for demineralization solution was also used in previous studies as an accelerated laboratory model [18]. Indeed, the local plaque pH could reach 4.5 or 4, supporting the use of the pH-cycling model to investigate remineralization [60].

Dentin hardness depends on the amount of calcified matrix per mm^2^ and the tubular density [61]. Hardness is an indirect measurement of mineral loss or gain in dental hard tissues [62]. Surface hardness recovery could provide evidence that remineralization occurred [27]. However, it has been reported that the hydration and dehydration of tissues may affect the mechanical properties of dentin [63,64]. Therefore, the mechanical properties of dentin should be evaluated on the fully hydrated tissues [63,64]. The cross-sectional hardness measured by nano-indentation may be more accurate to reflect dentin remineralization in the depth of dentin. In addition, elastic modulus is another important property of dentin that should be measured. Further study should measure the cross-sectional hardness and elastic modulus of demineralized and remineralized dentin using a nano-indentation method.

The results of pH and Ca and P ion release were similar to our previous study [17,65,66,67]. BMC rapidly increased the pH from 4 to above 5.5, and released large amount of Ca and P ions. The acid neutralization and Ca and P ion release of BMC should be attributed to the component of NACP. The control group showed a further demineralization and a decrease in root dentin hardness. In the present study, the remineralization of root dentin occurred during the 23-h immersion in artificial saliva at pH 7, and then the regenerated minerals were challenged by lactic acid solution at pH 4 for 1 h daily for 21 days. Therefore, the total remineralization effect depended on the rate of mineral regeneration in artificial saliva and the dissolution in lactic acid. It could be speculated that the demineralized root dentin alone could not induce effective remineralization during the immersion in artificial saliva due to its week nucleation ability, and the root dentin also could not resist the acid challenge because it did not have the acid-neutralization and Ca and P ion release capabilities. Hence, further demineralization occurred in root dentin in the control group. In addition, the further decrease in dentin hardness may also be partly attributed to the damage of the remaining collagen matrix in this severely demineralized outer zone. Dentin contains matrix metalloproteinases (MMPs) and cysteine cathespins, both of which could dissolve the collagen fibrils. These two types of enzymes may be activated as the dentin mineral is lost due to demineralization, thus degrading the collagen fibrils and further contributing to hardness loss [68,69].

The BMC group showed a moderate remineralization, with mineral precipitation in root dentin and a greater hardness than the control. BMC released Ca and P ions, leading to higher ion concentrations when immersed in artificial saliva, which would benefit the remineralization. Furthermore, when immersed in lactic acid at pH 4, BMC rapidly raised the pH to >5.5, and released a large amount of Ca and P ions, both of which could inhibit the demineralization of root dentin. However, the weak nucleation of root dentin limited the remineralization efficacy.

The PAMAM group had a remineralization effect similar to BMC group, although via a different mechanism. PAMAM macromolecules attracted Ca and P ions when immersed in artificial saliva, leading to rapid mineral regeneration. However, the remineralization effect of the present study, using a more realistic cyclic artificial saliva/lactic acid regimen, was much weaker than a previous study using constant pH 7 [37]. This indicates that remineralization tests performed at neutral pH overestimate the effects of a remineralization agent, because in vivo conditions have frequent low pH acidic challenges. Therefore, caution should be exercised in explaining remineralization studies performed at neutral pH.

In contrast, the novel BMC + PAMAM combined approach showed the most mineral regeneration and the greatest root dentin hardness, which reached the hardness of healthy root dentin. The combination of PAMAM with BMC achieved triple benefits of excellent nucleation templates, high Ca and P ion concentrations, and acid-neutralization. The results demonstrate the BMC + PAMAM method was effective in inhibiting root dentin demineralization and promote root dentin remineralization. It should be noted that the fast mineral deposition in the surface could lead to a hypermineralization phenomenon, which might prevent minerals from depositing into the deeper layers of dentin. Further study is needed to investigate whether hypermineralization occurred in the remineralization of demineralized dentin [70,71].

The novel BMC + PAMAM strategy could potentially be applied to various dental applications including, for example, root caries treatments. When root caries occurs, the Class V cavity could be filled with BMC, and then a PAMAM solution could be coated to cover the surrounding exposed root dentin, which achieved double benefits of demineralization inhibition and remineralization promotion. On the other hand, BMC is promising for Class V restorations to inhibit periodontal pathogens, combat periodontitis and protect the periodontium [10]. The combined use of BMC and PAMAM is beneficial for tooth root caries and protect tooth root structures, and is potentially applicable to Classes I, II and other types of tooth cavity restorations. Further studies are needed to investigate the novel BMC + PAMAM strategy in antibacterial and caries-inhibition tests for various dental restorations.

## 5. Conclusions

This study developed a novel bioactive multifunctional composite (BMC) for Class V restorations with a combination of remineralization, protein-repellent and antibacterial capabilities, and investigated the effects of this BMC and poly (amido amine) PAMAM on remineralization of demineralized root dentin in a cyclic artificial saliva/lactic acid for the first time. BMC + PAMAM was identified as the most effective method to inhibit root dentin demineralization and promote root dentin remineralization. BMC + PAMAM regenerated root dentin minerals and returned the pre-demineralized root dentin hardness to the level of healthy root dentin. The method of BMC + PAMAM is promising for a wide range of dental applications to combat caries and remineralize and protect tooth structures.
